# *In Silico* Prediction of Neuropeptides/Peptide Hormone Transcripts in the Cheilostome Bryozoan *Bugula neritina*

**DOI:** 10.1371/journal.pone.0160271

**Published:** 2016-08-18

**Authors:** Yue Him Wong, Li Yu, Gen Zhang, Li-Sheng He, Pei-Yuan Qian

**Affiliations:** 1 Division of Life Science, the Hong Kong University of Science and Technology, Clear Water Bay, Hong Kong, China; 2 Sanya Institute of Deep Sea Science and Engineering, Chinese Academy of Sciences, San Ya, Hai Nan, China; Universitat Wursburg, GERMANY

## Abstract

The bryozoan *Bugula neritina* has a biphasic life cycle that consists of a planktonic larval stage and a sessile juvenile/adult stage. The transition between these two stages is crucial for the development and recruitment of *B*. *neritina*. Metamorphosis in *B*. *neritina* is mediated by both the nervous system and the release of developmental signals. However, no research has been conducted to investigate the expression of neuropeptides (NP)/peptide hormones in *B*. *neritina* larvae. Here, we report a comprehensive study of the NP/peptide hormones in the marine bryozoan *B*. *neritina* based on *in silico* identification methods. We recovered 22 transcripts encompassing 11 NP/peptide hormone precursor transcript sequences. The transcript sequences of the 11 isolated NP precursors were validated by cDNA cloning using gene-specific primers. We also examined the expression of three peptide hormone precursor transcripts (BnFDSIG, BnILP1, BnGPB) in the coronate larvae of *B*. *neritina*, demonstrating their distinct expression patterns in the larvae. Overall, our findings serve as an important foundation for subsequent investigations of the peptidergic control of bryozoan larval behavior and settlement.

## Introduction

The bryozoan *Bugula neritina* is a cosmopolitan marine fouling organism. *Bugula neritina* are colonial and have a complex life cycle that consists of a rather short free swimming larval phase followed by a sessile adult stage [1]. *B*. *neritina* produces coronate larvae, an oval-shaped type of larva with a ciliated epithelium, a pair of simple photosensory eyespots, an undifferentiated apical cell mass (the apical blastema), an internal sac, and a pyriform organ. All these major larval tissues are innervated and connected by the larval nervous system [2]. Prior to the initiation of permanent attachment, the coronate larvae have a relatively short pelagic phase for substratum exploration. Metamorphosis can be triggered by both endogenous and environmental signals [3]. It is generally believed that the induction of larval settlement in *B*. *neritina* is a neuronally regulated process. For example, previous studies have demonstrated that different neurotransmitters can be either inductive or inhibitory to larval settlement of *B*. *neritina* [4, 5]. The pyriform organ, apical disc and ciliated epithelial layer have been suggested to play a sensory role during the exploration process [6]. During metamorphosis, *B*. *neritina* larvae undergo dramatic morphological and structural transformations [3]. Following a series of morphological changes during the initial phase of metamorphosis, the larva permanently attaches onto the substratum, and the larval body is re-organized. Reed and Woollacott have demonstrated that substantial muscular contractions initiate the initial morphogenetic process [7]. It is clear that a well-designed neural circuit precisely regulates the musculature-mediated structural re-arrangement. Therefore, a comprehensive understanding of the larval neural element is necessary.

Neuropeptides (NP) are small peptides that are produced by neurons and are generally less than 50 amino acids in length. After being synthesized in the cell body of neurosecretory cells, they are transported to a specific location along the neurite, such as the synaptic junction, and are released from the neurite in response to neural stimulation. Their action depends on the expression of the corresponding receptor in target cells [8]. NP are the largest and most diverse class of signaling molecules and are involved in the regulation of many physiological functions [9]. The diversity of NPs varies in different taxa. In past decades, major efforts have focused on the discovery and functional characterization of NPs in different animal phyla [10, 11]. The conventional NP identification process depends on bioassay-guided discovery. Although this approach enables the separation and identification of several classes of classical NPs, a substantial amount of starting material, namely the protein sample from the neuron, is needed. Consequently, this technique is limited to animals such as decapods or insects, from which sufficient protein can be extracted for peptide fractionation and mass spectrometry identification [12]. With the advance of new technologies, direct imaging mass spectrometry (IMS) has become a powerful and specific technique for the discovery of NPs during the last decade [13–15]. The limitation of the MS technique, however, is that the accuracy of mass spectrometry is critical. Recently, the development of high-throughput next-generation sequencing technology [16–18], as well as increasing public availability of expressed sequence tag (EST) libraries, has enabled an alternative approach to the discovery of NPs. Briefly, homology searches using known NP peptide sequences were performed with relatively unrestrictive matching criteria to avoid the exclusion of candidate NP transcripts from the EST library. Candidate genes were then filtered based on a number of NP hallmark characteristics, such as the presence of the N-terminal signal peptide, dibasic cleavage sites, and prediction of the mature NP [19]. In conjunction with online software programs, it is possible to predict the post-translational processing of the deduced peptides. In short, *in silico* genome/transcriptome mining has been shown to be a successful and effective method for the discovery of NPs [19–21].

Recently, a transcriptome database of various stages of metamorphosis of *B*. *neritina* was constructed [22]. This database provided the foundation for *in silico* transcriptome mining of NP expressed in *B*. *neritina* larvae. Here, we aimed to provide the first comprehensive report of peptide hormones in *B*. *neritina* larvae using *in silico* transcriptome mining. We identified 22 transcripts encompassing 11 NP precursor sequences. The expression of 11 NP precursors was validated by cDNA cloning. We successfully identified the spatial expression patterns of three NP transcripts in the coronate larva of *B*. *neritina*. This is the first neuropeptidome study of the bryozoan *B*. *neritina*.

## Materials and Methods

### Larval sample preparation

Adults from *B*. *neritina* colonies were collected from the floating rafts and other submerged objects at a fish farm in Trio Bench, Hong Kong (22°21′19 N, 114°16′15 E) from winter 2013 to spring 2015 and then maintained in an aquarium at 21°C with a flow-through seawater system at the Coastal Marine Laboratory of Hong Kong University of Science and Technology for less than seven days before use. Permission to collect *B*. *neritina* colonies from the fish farm was provided by the fish farm owner. Prior to larval release, adult colonies were transferred to a transparent fish tank containing 10 L of 0.22-μm filtered seawater. Spawning of free swimming larvae were triggered and concentrated by a point source illumination. For in situ hybridization, larval specimens were fixed in 4% paraformaldehyde in 0.22-μm autoclaved filtered seawater (AFSW). For RNA extraction, approximately 1000 larvae were transferred to a 1.5-mL Eppendorf tube. After removal of residual sea water, larval specimens in the tube were immediately transferred to a liquid nitrogen storage tank. RNA samples from larval specimens were extracted two months after cryogenic storage.

### Transcriptome mining of NPs/peptide hormone precursors

The transcriptome database generated from our previous study was used to screen for NP hormone candidate transcripts. Database searches to discover NPs/peptide hormones were conducted using methods that were modified according to recent publications [19, 23–27]. Protein sequences of the known NPs and peptide hormones in invertebrates were downloaded from Uniprot Knowledgebase (http://www.uniprot.org/) using “NP”, “hormone” or “neurohormone” as search keywords without “receptor”, “receptor associated”, “hypothetical”, “uncharacterized”, “signal”, “cytochrome”, “esterase”, “transferase”, “binding protein”, “hydrolase”, “lipase”, “inducible protein”, “methyltransferase” or “transmembrane”. The screened sequences were transformed into FASTA format to generate a local peptide database. The transcriptome of *B*. *neritina* was blasted against the known peptide database mentioned above in tblastn mode to mine for putative cDNA sequences encoding NPs/peptide hormones. For each peptide query, the first three with the lowest E-value (less than 0.01) were chosen as candidates and manually checked for homology to known peptides.

### Prediction of mature peptide structures

The structures of the selected candidate sequences were predicted based on previously established procedures [19]. These sequences were translated using the ExPASy translate tool (http://www.expasy.org/). Each deduced peptide was assessed for typical NP precursor features, including start and stop codons, the presence of an N-terminal signal peptide sequence, and pro-hormone convertase processing sites [28]. Signal peptide prediction was conducted using the online program SignalP 4.1 Server [29]. Pro-hormone cleavage sites were predicted based on the information supplied by Veenstra [30] and the Neuropred online program (http://neuroproteomics.scs.illinois.edu/cgi-bin/neuropred.py), as well as homology to known NP precursors. The web-based software DiANNA 1.1 was used to predict disulfide bridges in putative mature peptide sequences [31]. The online program Sulfinator was used to predict the sulfation state of Tyr residues [32]. In some cases, disulfide bridge formation and other post-translational modifications, e.g., cyclization of N-terminal Glu/Gln residues and C-terminal amidation at Gly residues, were predicted mainly based on homology to known peptide isoforms [24].

### RNA extraction and cDNA synthesis

Total RNA was extracted from the free-swimming stage, the early pre-ancestrulae stage, the mid pre-ancestrulae stage and the juvenile stage of *B*. *neritina* using TRIzol^®^ reagent (Invitrogen, Carlsbad, CA, USA) according to the standard protocol provided by the manufacturer. To remove DNA contaminants, total RNA was treated with a TURBO DNA-freeTM kit (Ambion Inc., Austin, TX, USA). First-strand cDNA was prepared from the total RNA from each stage using M-MLV reverse transcriptase (USB^®^ Product, Cleveland, OH, USA) with oligo(dT) primers [33].

### RACE sequencing and peptide confirmation

Because NP precursor sequences generated from the transcriptome are often fragmented or incomplete, the rapid amplification of cDNA ends (RACE) technique was performed to obtain full-length open reading frames (ORFs) of the predicted genes for further confirmation. Two sets of gene-specific primers were designed based on the partial cDNA sequences from the *B*. *neritina* transcriptome database. The sequences of the reads and the corresponding primers are listed in S1 Table. For 5′ RACE, an oligo(dC) tail was added to the 3′ cDNA ends of the cDNA template by terminal deoxynucleotidyl transferase (TdT) (USB, Cleveland, Ohio, United States). Gene-specific reverse primers and an oligo(dG) (10) adaptor primer were used to perform nested PCR. The 3′ RACE was performed in the same manner as described for 5′ RACE, except an oligo(dT) adaptor primer and gene-specific forward primers were used. The primer sequences are listed in S1 Table. PCR products were gel-purified and then ligated into the pMD18-simple T vector (TaKaRa Bio Inc., Dalian, China), transformed and sequenced (BGI Company, China). NCBI Blastx was performed to check the homology between the full-length ORFs and the predicted NP/neurohormone amino acid sequences. If a cloned sequence did not match the predicted NP/neurohormone, it was excluded. All NP precursor transcript sequences were submitted to NCBI GenBank, and the accession numbers are shown in Table 1.

**Table 1:** Neuropeptide/peptide hormones predicted on the basis of transcriptome mining of *Bugula neritina*.

Name	Peptide family	Contig No. (From *B*. *neritina* transcriptome)	Transcript length (bp)	Reference accession No.	5' RACE confirmed	3' RACE confirmed	*E*-value	Score	GenBank accession No.
BnVP-like	Arginine-vasotocine	BN_contig_97286, BN_contig_87480	599	AAX18227.1	+	+	4.00E-09	61.2	KX095876
BnPEP-like	Pedal peptide/orcokinin neuropeptide	BN_contig_141741, BN_contig_166562, BN_contig_98929	792	AHB62384.1	+	-	1.00E-05	55.1	KX095877
BnFDSIG-like	Pedal peptide/orcokinin neuropeptide	BN_contig_220841 BN_contig_55496	697	AEE25644.1	+	+	1.00E-10	72	KX095878
BnNPF-like	Neuropeptide Y	BN_contig_222301, BN_contig_184693	476	AGM46557.1	+	+	8.00E-10	61.6	KX095879
BnGHB	Glycoprotein hormone-beta5-I	BN_contig_4814	949	CAR95348.2	+	+	6.00E-08	57.4	KX095880
BnILP-A	Insulin-like peptide	BN_contig_62648, BN_contig_127297	1220	XP_008642491.1	+	+	3.00E-27	112	KX095882
BnINS_B	Insulin-like peptide	BN_contig_36942	371	Q9W7R2.1	-	-	4.00E-25	44.3	KX095885
BnINS_C	Insulin-like peptide	BN_contig_203950	675	CAC20109.1	+	+	3.00E-04	59.7	KX095886
BnILP-D	Insulin-like peptide	BN_contig_236733	1029	XP_011415724.1	+	+	5.00E-08	59.3	KX095881
BnILP-E	Insulin-like peptide	BN_contig_208700, BN_contig_236692	696	XP_012388351.1	+	-	1.00E-11	69.3	KX095883
BnILP-F	Insulin-like peptide	BN_contig_8730	547	ABB23272.1	-	+	5.00E-17	83.6	KX095884

### Sequence analysis

Corresponding NP sequences were downloaded from the NCBI protein sequence database, and the known NP genes and proteins were compiled. Predicted mature peptide sequences together with reference sequences were used for the alignment using the bioinformatics software Mega 6.0 [34].

### In situ hybridization (ISH) (whole-mount) and section ISH (SISH)

Each target NP gene transcript was amplified using the transfected vector carrying the target gene fragment acquired by 3’ RACE, and the corresponding gene-specific forward and reverse primers are listed in S1 Table. The T7 promoter sequence was conjugated to the 5’ end of the gene-specific reverse primer (S1 Table). The anti-sense digoxin-labeled probes were synthesized using the DIG RNA labeling kit (Roche Diagnostics, Nutley, NJ, USA). Swimming larvae were collected and fixed in freshly prepared 4% paraformaldehyde overnight at 4°C. The fixed samples were then washed three times with PBS to remove the fixative, dehydrated with 100% methanol and stored at -20°C until further processing. The experiment was conducted based on a previously described method [35] with minor modifications. The samples were rehydrated by sequential dilution series of methanol in PBS. The samples were washed three times with PBST (PBS-0.1% TritonX100) and then permeabilized with 10 μg/mL of proteinase K (Invitrogen) in PBST at room temperature for 7 minutes. The specimens were post-fixed in 4% PFA and then washed five times with PBST. After pre-hybridization at 56°C for 1 hour in a hybridization mix, RNA probe hybridization was performed at 56°C overnight. Washing was conducted with pre-warmed hybridization mix (without heparin and salmon sperm DNA) using sequential concentrations followed by three washes with PBST. Prior to immunostaining, the hybridized samples were incubated with blocking solution (4% BSA in PBST) for 1 hour at room temperature. The samples were then incubated with anti-DIG-AP antibody (Roche Applied Science, Indianapolis, IN) at a concentration of 1:5000 in blocking solution at 4°C overnight with gentle rotation. Next, the samples were washed six times with PBST and then incubated in alkaline Tris buffer (0.1 M Tris-HCl pH 9.5, 50 mM MgCl_2_, 0.1 M NaCl, 1% Tween-20) containing NBT and BCIP (Promega) at room temperature in the dark. The samples were washed with stop solution until a deep blue or purple color developed. After mounting with 80% glycerol, the specimens were observed using a light microscope (Olympus, Tokyo, Japan). Because the whole-mount samples were not completely transparent, paraffin sectioning of the hybridized samples was performed to visualize the internal expression pattern.

## Results and Discussion

### NPs and peptide hormones predicted in *Bugula neritina*

In this study, putative NP-encoding transcripts were predicted *in silico* from the *B*. *neritina* transcriptome reported in a previous study, and then verified by molecular cloning. This is the first study to attempt to identify NP and peptide hormones in bryozoan species. In summary, 11 NP precursor sequences were predicted, and 10 of them were confirmed by transcript cloning (Table 1).

**Fig 1:**
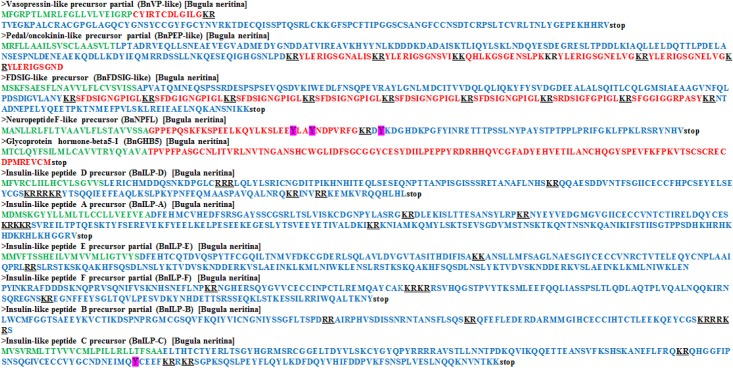
Precursor sequences of neuropeptide/peptide hormones from *Bugula neritina*. Green: signal peptide; Red: putative mature peptide; Blue: preprotein sequence; Black underline: dibasic cleavage site.

Because many NPs are relatively fragmented in the transcriptome dataset, the BLAST scores tended to be low (with a high E-value). This issue has been reported in previous studies and was not critical for the application of *in silico* methods [19]. Full-length cloning of NP transcripts was used to confirm the *in silico* predictions. The NP precursor sequences and predicted structures are listed in Table 1 and Fig 1, respectively.

The predicted NP transcript precursors identified in this study included six insulin-like peptides (BnILP-A, BnILP-B, BnILP-C, BnILP-D, BnILP-E, BnILP-F), two pedal peptide-like sequences, BnPedal/orcokinin-like (BnPEP-like) and BnFDSIG-like, one vasopressin-like peptide sequence (BnVP-like), one NP F-like sequence (BnNPF) and glycoprotein hormone-beta 5-I (BnGPB) (Table 1). Multiple sequence alignment analysis clearly demonstrated that the deduced protein sequences of BnVP-like, BnNPF, BnGHB and all six BnILP precursor transcripts were homologous to known NPs (Table 2, details discussed below). Their presence in the *B*. *neritina* transcriptome requires further confirmation. Although the spatial localization of the RYamide and FMRFamide NPs has been examined by immunohistochemistry in previous studies [36, 37], the transcripts of these two NPs were not detected in our study. NP production occurs mainly in the cell body of neurosecretory cells. In *B*. *neritina* swimming larvae, however, the nervous system is rather simple, comprising a small number of neurons and, potentially, only a few neurosecretory cells [2]. Thus, NP transcripts may be present at relatively low abundances in the transcriptome library prepared from whole swimming larvae. Rather low coverage of the NP transcripts was expected. The NP transcripts were rather fragmented and could not be recovered using our *in silico* prediction method. We predicted that the RYamide and FMRFamide NP transcripts were expressed but at very low abundances; therefore, they could not be recovered from the transcriptome. Relatively low transcript abundance may also explain our high failure rate (8 out of 11) in detecting the expression of NP precursor transcripts by in-situ hybridization. To improve the recovery rate of NP transcripts, the most direct strategy is to increase the coverage depth of the *B*. *neritina* larval transcriptome. Deep coverage of the transcriptome has the advantage of facilitating the recovery of full-length transcripts and of increasing the possibility of resolving transcripts with a low abundance. However, this approach requires much higher sequencing costs and computational power. An alternative technique would be to isolate the neurons from swimming larvae and perform a transcriptomic analysis of the isolated neurons. Okano et al. has developed an enzymatic cocktail to dissociate neurons from *B*. *neritina* swimming larvae [38]. The development of a technique to isolate *B*. *neritina* larval neurons may enable high-resolution transcriptome profiling and facilitate the discovery of novel NP transcripts.

**Table 2:** Predicted neuropeptide/peptide hormones from *Bugula neritina*.

Precusory transcript Name	Predicted mature peptide (variant) name	Predicted mature peptides
BnFDSIG-like	P1	SFDSIGNGPIGLKR
	P2	SFDGIGNGPIGLKR
	P3	SRDSIGFGPIGLKR
	P4	SFGGIGGRPASYKR
BnPep-like	P1	YLERIGSGNALISKR
	P2	YLERIGSGNSVIKK
	P3	QHLKGSGENSLPKKR
	P4	YLERIGSGNELVGKR
	P5-partial	YLERIGSGND
BnVP-like	Vasopressin-like	CYIRTCDLGILGKR
	Neurophysin	TVEGKPALCRACGPGLAGQCYGNSYCCGYFGCYNVRKTDECQISSPTQSRLCKKGFSPCFTIPGGSCSANGFCCNSDTCRPSLTCVRLTNLYGEPEKHHRV
BnGHB5		TPVPFPASGCNLITVRLNVTNGANSHCWGLIDFSGCGGYCESYDIILPEPPYRDRHHQVCGFADYEHVETILANCHQGYSPEVFKFPKVTSCSCRECDPMREVCM
BnNPFL		GPPEPQSKFKSPEELKQYLKSLEEY(SOH)LAY(SOH)NDPVRFGKR
BnILP-A	ChainB	DFEHMCVHEDFSRSGAYSSCGSRLTSLVISKCDGNPYLASRGKR
	ChainC	DLEKISLTTESANSYLRPKR
	ChainA	NYEYVEDGMGVGIICECCVNTCTIRELDQYCESKR
BnILP-B	ChainB	GTSAEEYKVCTIKDSPNPRGMCGSQVFKQIYVICNGNIYSSGFLTSPDRR
	ChainC	AIRPHVSDISSNRNTANSFLSQSKR
	ChainA	QFEFLEDERDARMMGIHCECCIHTCTLEEKQEYCGSKR
BnILP-C	ChainB	TFSAAELTHTCTYERLTSGYHGRMSRCGGELTDYVLSKCYGYQPYRRRR
	ChainC	AVSTLLNNTPDKQVIKQQETTEANSVFKSHSKANEFLFRQKR
	ChainA	QHGGFIPSNSQGIVCECCVYGCNDNEIMQYCEEFKR
BnILP-D	ChainB	LERICHMDDQSNKDPGLCRRRLQLYLSRICNGDITPIK
	ChainC	HNHITEQLSESEQNPTTANPISGISSSRETANAFLNHSKR
	ChainA	QQAESDDVNTFSGIICECCFHPCSEYELSEYCGSKRRRKR
BnILP-E	ChainB	DFEHTCQTDVQSPYTFCGQILTNMVFDKCGDER
	ChainC	LSQLAVLDVGVTASITHDIFISAKK
	ChainA	ANSLLMFSAGLNAESGIYCECCVNRCTVTELEQYCNPLAAIQPRLRR
BnILP-F	ChainB (partial)	PYINKR
	ChainC	AFDDDSKNQPRVSQNIFVSKNHSNEFLNPKR
	ChainA	NGHERSQYGVVCECCINPCTLREMQAYCAKKR

### NP sequences and spatial expression pattern analysis

#### FDSIG NP and Pedal/orcokinin-like NP transcripts

**Fig 2:**
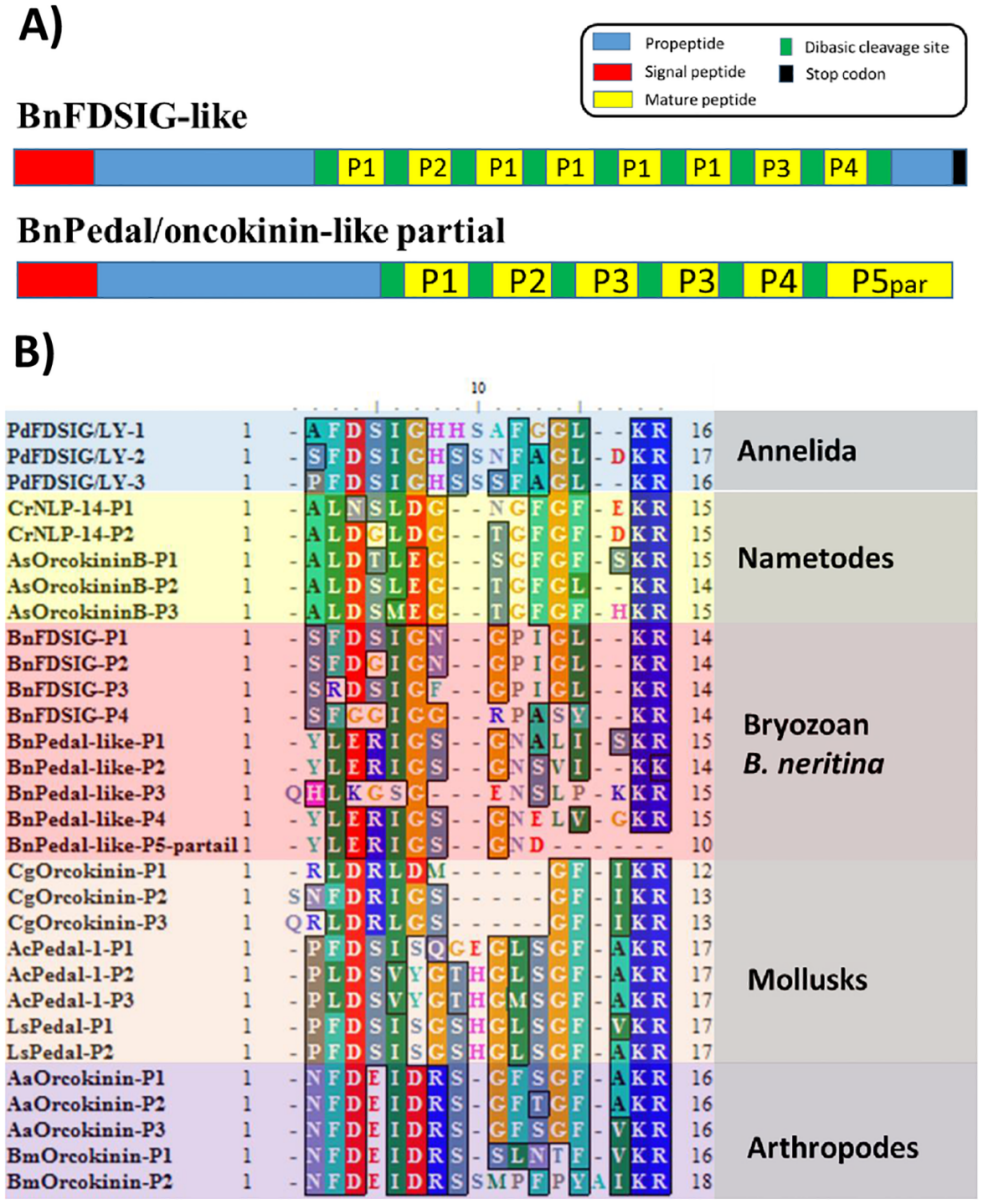
A) Primary structure of the deduced precursor sequence of BnFDSIG-like and BnPedal/orcokinin-like and B) multiple sequence alignment of the mature FDSIG and Pedal/orcokinin neuropeptides. Mature peptide sequences were derived from precursor protein sequences download from NCBI GenBank. PdFDSIG-P1-3: (AAP57098.1) pedal peptide preprohormone [*Lymnaea stagnalis*]; CrNLP-14-P1-3: (EFO91279.1) CRE-NLP-14 protein [*Caenorhabditis remanei*]; AsOrcokininB-P1-3: (ERG84279.1) orcokinin peptides type b [*Ascaris suum*]; CgOrcokinin-P1-3: (XP_011447518.1) PREDICTED: orcokinin peptides type A-like [*Crassostrea gigas*]; AcPedal-1-P1-3: (NP_001191585.1) pedal peptide-1 precursor [*Aplysia californica*]; LsPedal-P1-2: (AAP57098.1) pedal peptide preprohormone [*Lymnaea stagnalis*]; AaOrcokinin-P1-3: (AFK81939.1) orcokinin [*Amphibalanus amphitrite*]; BmOrcokininP1-2: BAG50371.1| orcokinin [*Bombyx mori*].

Two pedal peptide/orcokinin NP precursor transcripts, BnFDSIG-like and BnPep-like, were predicted. BnFDSIG-like consisted of two partial transcripts in the transcriptome reference base (Table 1). These two transcripts were aligned with the LYamide/FDSIG NP and pedal peptide precursor 2 from the marine polychaete *Platynereis dumerilii* [39], respectively. The full-length BnFDISG-like transcript was acquired by 5’ and 3’ RACE. The deduced protein precursor of the BnFDISG transcript encodes a 293-amino-acid protein, in which the first 24 amino acids are the signal peptide. *In silico* cleavage of the predicted precursor protein yields eight mature peptides with 12 amino acids comprising four peptide variants (P1/2/3/4) (Fig 2A, peptide sequences listed in Table 2). Similar to the P. *dumerilii* LYamide/FDSIG NP, the predicted mature peptide variant P1 contains the C-terminal SFDSIG motif, whereas the other three peptide variants possess one or two amino acid substitutions in the FDSIG motif, such as SFDGIG in P2, SRDSIG in P3 and SFGGIG in P4 (Fig 2B, Table 2). While these putative mature peptides of BnFDSIG-like greatly resembled the FDSIG motif of the *P*. *dumerilii* LYamide/FDSIG NP, they lacked the LYamide motif (Fig 2B). The predicted BnFDSIG peptide variants P1, P2 and P3 exhibited a basic SxDxIGxGPIGL structure, whereas variant P4 shared little similarity with the other three variants (Fig 2B).

For another pedal peptide precursor transcript, BnPedal/orcokinin-like, although we were unable to reach the 3’ end of the transcript, *in silico* prediction of the deduced precursor partial protein sequence yielded at least six mature peptides (one partial sequence) with five peptide variants constituting 12–14 amino acid residues (peptide sequences listed in Table 2). The predicted mature peptides of BnPedal/orcokinin-like exhibited a basic C-terminal YLERIGSGNxxx(x)-NH2 motif structure. The predicted mature peptides of BnFDSIG-like and BnPedal/orcokinin-like shared a C-terminal xxxxIGxGxxx structure (IGxG motif highlighted in the purple square in Fig 2B). Instead of a FDSIGxGP motif, the predicted mature peptides of BnPedal/orcokinin-like exhibited a LERIGSGN motif in the counterpart region (Fig 2B). Such motif structures exhibited remarkable similarity to orcokinin peptides from the Pacific Oyster *Crassostrea gigas*, which exhibited a (F/L)DRLGSG motif (Fig 2B). Interestingly, *C*. *gigas* pedal peptides shared much less similarity to the pedal peptides from the molluscan *Aplysia californica* and *Lymnaea stagnalis* compared with the putative BnPedal/orcokinin-like peptides (Fig 2B).

**Fig 3:**
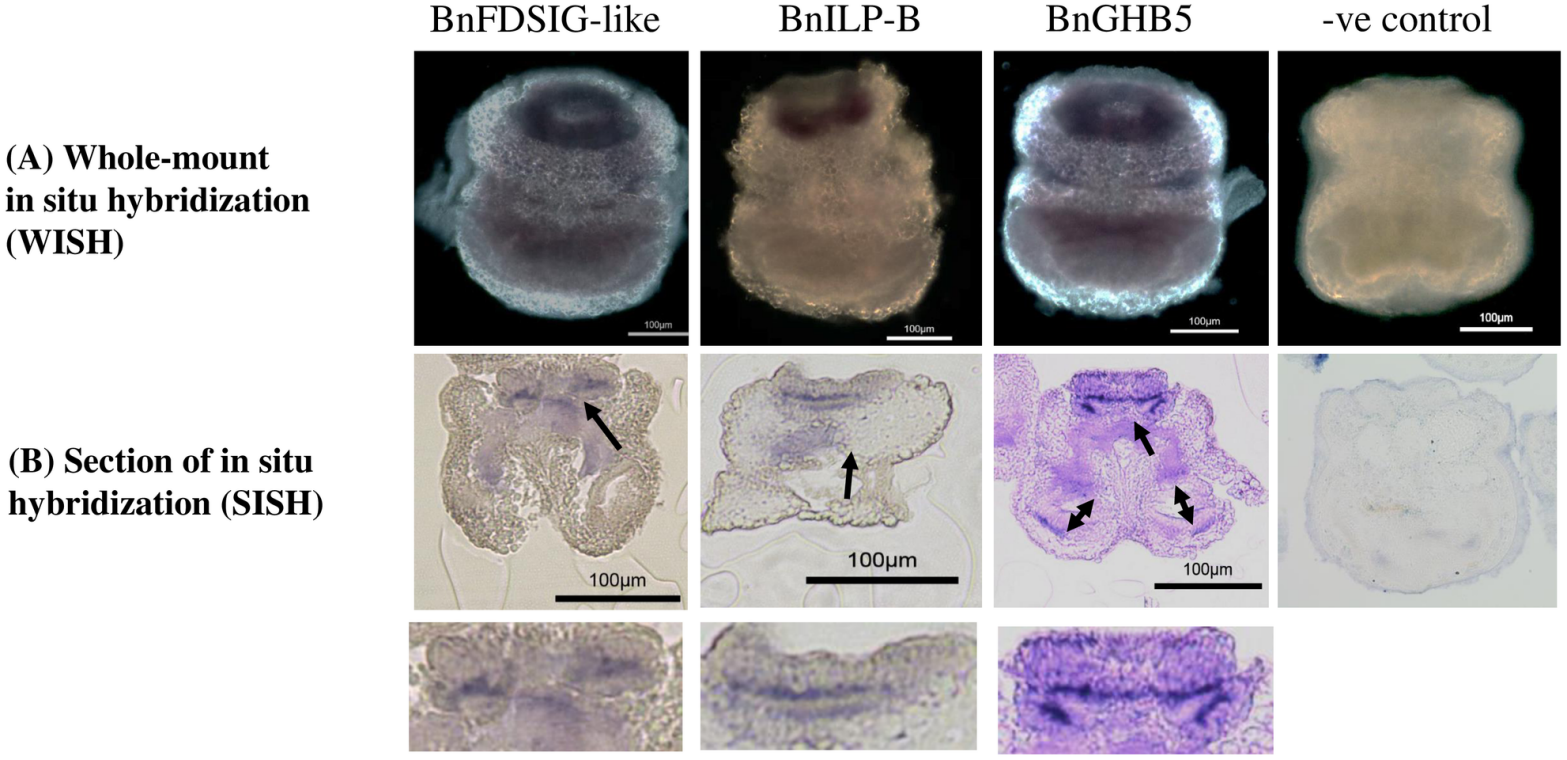
Expression of BnFDSIG-like, BnILP-B, and BnGHB5 during the larval stage. (A) Whole-mount *in situ* hybridization (WISH), (B) a representative section of WISH and C) enlarged image of the region highlighted by the red dotted line in B), which includes the apical organ and neural plexus.

Pedal peptide/orcokinin (PP/OK)-type peptides are a family of structurally related NPs. Pedal peptides were first isolated from the pedal ganglion of *Aplysia californica* [40]. However, orcokinin was initially isolated from the abdominal nerve cord of the crayfish *Orconectus limosus* [41]. PP/OK-type peptides have been attributed a range of functions in different taxa, including myoactivity, seasonal physiology and endocrine regulation in arthropods, locomotion in both annelids and mollusks [40, 41, 42 43], and circadian regulation in the cockroach *Leucophaea maderae* [44–46]. Currently, nothing is known about the physiological functions of PP/OK-type peptides in bryozoans. Whole-mount *in situ* hybridization (WISH) and sections of WISH (Fig 3) of the BnFDSIG-like precursor transcript revealed expression at the junction between the epidermal and mesodermal blastemal layer of the apical organ as well as at the neuromuscular junction in the root region of the internal sac (Fig 3A & 3B). The BnFDSIG-like precursor transcript was also strongly expressed in the apical organ, with much weaker signal detected at the equatorial nerve cord and in the internal sac of both the epidermal and mesodermal blastemas (Fig 3A & 3B). The equatorial nerve cord was suggested to play both a sensory and locomotor role in swimming larvae [47]. While neurites from the equatorial nerve cord direct innervate ciliated corona cells, innervation of intercoronal sensory cells has also been reported [47]. Expression of the BnFDSIG-like precursor transcript in the equatorial nerve ring (Fig 3A) suggested the involvement of BnFDSIG-like peptides in locomotion in *B*. *neritina* larvae. The absence of BnFDSIG-like precursor transcript expression in the centric part of the apical organ as well as the centric part of the larvae suggested that these peptides were not produced by the anterior nerve cord, which connects the sensory apical radical ciliated cells to the central nerve ring [2, 47]. The role of BnFDSIG-like precursor transcript expression in the junction between the epidermal and mesodermal blastemal layer of the apical organ is currently unclear.

### Glycoprotein hormone

**Fig 4:**
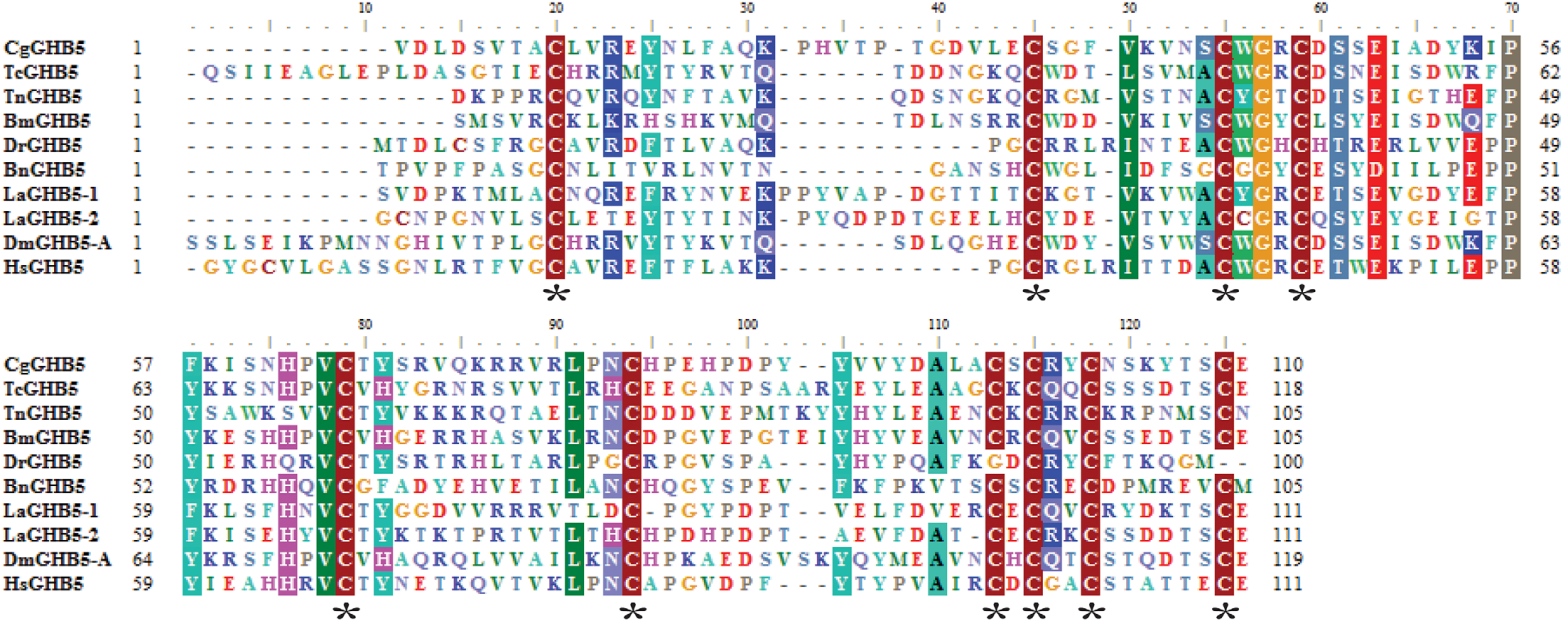
Sequence alignment of the *Bugula neritina* putative mature glycoprotein hormone beta 5 with homologs. CgGHB5: (EKC24490.1) glycoprotein hormone beta 5 *Crassostrea gigas*; TcGHB5: *(*EFA10760.1glycoprotein hormone beta 5) *Tribolium castaneum;* TnGHB5: (KRZ59516.1) glycoprotein hormone beta 5 *Trichinella nativa;* BmGHB5: (NP_001124380.1) glycoprotein hormone beta 5 *Bombyx mori;* DrGHB5: (XP_003198997.1) glycoprotein hormone beta 5 *Danio rerio;* LaGHB5-1 (XP_013400795.1) glycoprotein hormone beta 5-like isoform 1 *Lingula anatina;* LaGHB5-2: (XP_013400794.1) glycoprotein hormone beta 5-like isoform 2 *Lingula anatina*; DmGHB5: (NP_001104335.1) glycoprotein hormone beta 5 *Drosophila melanogaster;* HsGHB5: (AAO33390.1) glycoprotein hormone beta 5. *Homo sapiens*. The asterisk “*” marks the conserved cysteine that will form dimers.

**Fig 5:**
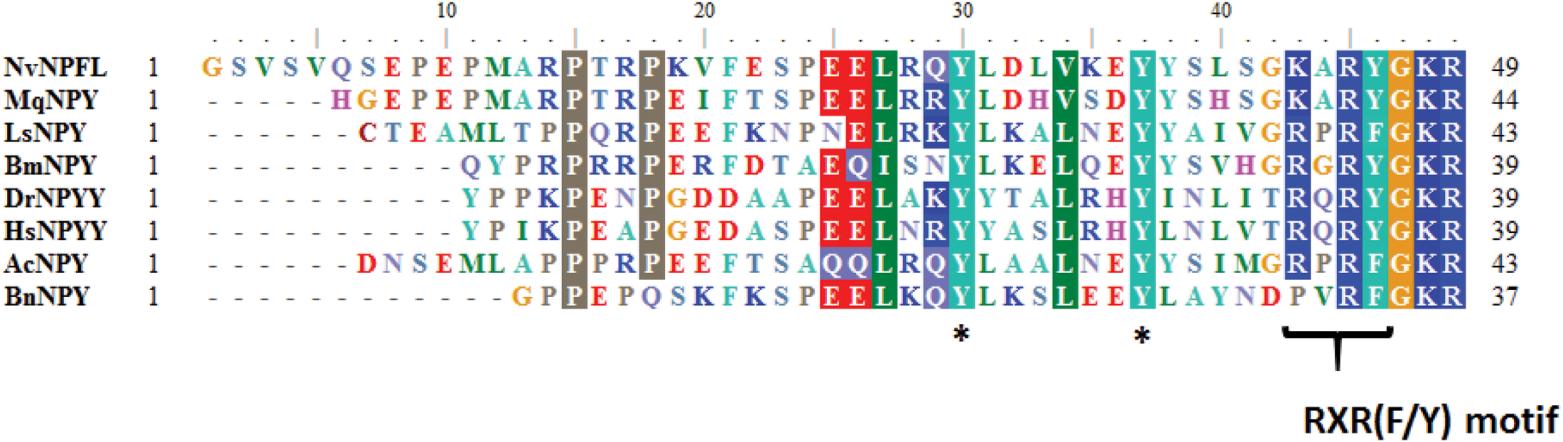
Sequence alignment of *Bugula neritina* putative mature Neuropeptide F with homologs. AcNPY: (NP_001191635.1) pro-neuropeptide Y precursor *Aplysia californica*; HsNPYY: (CAG46926.1) NPYY *Homo sapiens*; LsNPY: (CAB63265.1) NPY *Lymnaea stagnalis*; NvNPFL: (XP_008206766) NPF *Nasonia vitripennis;* DmBPYY: *(*AGB96023.1) NPF *Drosophila melanogaster*; BmNPY: *(*NP_001124361.1) NPF *Bombyx mori*. The RXR(F/Y) modified are conserved except in the putative mature peptide of BnNPY. The asterisk “*” denotes the conservation of tyrosine (Y) residues that are predicted to be sulfated in the mature peptide in the NPF family.

One putative glycoprotein hormone-β5 (GPB5) transcript was predicted by *in silico* prediction (Table 1). The full-length transcript exhibited weak but significant homology to GPB5 in *Bombyx mori*. This transcript encoded a 130-amino-acid protein, in which the first 24 amino acids were the predicted signal peptide (S1 Table). No dibasic cleavage sites were detected in this hormone precursor. Alignment with glycoprotein hormone beta homologs from vertebrates and invertebrates revealed nine highly conserved Cys residues (Fig 4). The GPB5 found in *B*. *neritina* contained conserved Cys residues, which likely form a cysteine knot structure in common with other glycoprotein hormone subunits [48]. The results of the glycoprotein hormone WISH (Fig 3A) and a section of WISH (Fig 3B) are shown in Fig 5. The spatial gene expression patterns of BnGPB5 were similar to those of BnFDSIG-like. However, this hormone was also expressed in the wall region of the internal sac (Fig 3B).

The glycoprotein hormone is highly homologous to other cysteine knot growth factor (CKGF) families, including bursicon, which is well known for its effects on cuticle tanning/melanization during the ecdysis process in insects [49]. In *B*. *neritina*, the spatial gene expression patterns of GPB were detected in the apical organ and the wall region of the internal sac, which form the epidermis of the body wall and secrete a cuticular exoskeleton that undergoes calcification [6]. These observations suggest that BnGPB5 might be involved in the development of the adult body wall.

### Insulin-like peptides

The insulin family comprises several members, including insulin, insulin-like growth factor (IGF) and relaxin in vertebrates [50–52], insulin-like peptide in insects [53] and the molluscan insulin-related peptide (MIP) in mollusks. Insulin in invertebrates is produced by neuroendocrine cells in the central nervous system (CNS) [54]. Classical insulin precursor contains contiguous B-C-A peptides, in which the A- and B-chain peptides are linked together by three disulfide bonds after maturation. The C-chain peptide assists the formation of the linkage and is subsequently removed from the cleavage site [55].

Six putative insulin-like peptide precursor transcripts (BnILP-A, BnILP-B, BnILP-C, BnILP-D, BnILP-E and BnILP-F) were found in *B*. *neritina*. Among these genes, the full-length sequences of BnILP-A, BnILP-C and BnILP-D were isolated and sequenced using RACE (Table 1). Although only two of the six putative insulin-like peptide precursor transcripts covered the full ORF, multiple sequence alignment with insulin and insulin-like peptides homologs revealed a clear B-C-A chain peptide structure in all six putative mature insulin-like peptides, with a putative dibasic cleavage site at the junction between each putative chain (Fig 5). All six putative insulin-like peptide were predicted to form four cysteine bridges (Fig 5). The WISH results localized BnILP1 to the blastemal of the apical organ (Fig 3), similar to the results obtained for BnFDSIG and BnGPB5. During metamorphosis the apical organ undergoes substantial development. We speculate that BnILP-A may regulate the development of the polypide during metamorphosis.

### Neuropeptide F

**Fig 6:**
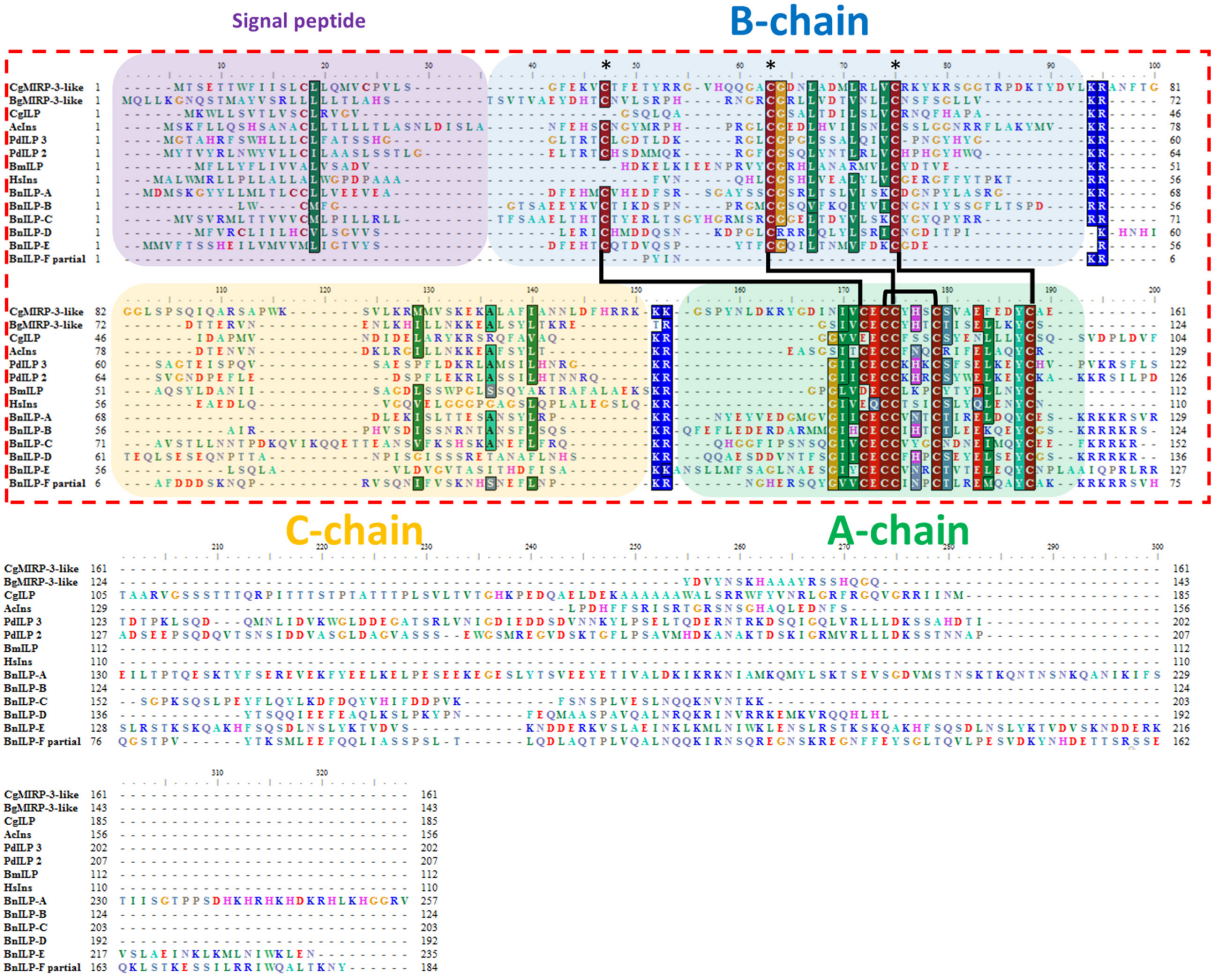
Sequence alignment of the *Bugula neritina* insulin-like peptide precursor protein with homologs. CgMIRP-3-like: (XP_011415724.1) PREDICTED: molluscan insulin-related peptide 3-like [*Crassostrea gigas*]; BgMIRP-3-like: (XP_013070273.1) PREDICTED: molluscan insulin-related peptide 3-like [*Biomphalaria glabrata*]; CgILP: (XP_011455161.1) PREDICTED: insulin-like peptide [*Crassostrea gigas*]; AcIns: (AF160192_1) insulin precursor [*Aplysia californica*]; PdILP 3: (AHB62360.1) insulin-related peptide 3 neuropeptide precursor [*Platynereis dumerilii*]; PdILP 2: (AHB62359.1) insulin related peptide 2 neuropeptide precursor [*Platynereis dumerilii*]; HsIns: (AAA59172.1) insulin [*Homo sapiens*]. Putative monobasic/dibasic cleavage sites are indicated by blue blocks. Amino acid residues corresponding to signal peptide. The A chain, B chain and C chain are indicated by a purple, green, blue and orange background color, respectively. Disulfide bonds are indicated by black lines connecting the two corresponding cysteine residues.

Neuropeptide F (NPF) was first identified in the tapeworm *Moniezia expansa* [56], and it was considered to be homologous to neuropeptide Y in vertebrates. Mature NPFs are typically 36 amino acids long and possess the C-terminal motif–RXRFamide (commonly RPRFa) and two tyrosine residues at positions 10 and 17 from their C-termini [57]. Two neuropeptide F-like transcripts were recovered from the *B*. *neritina* transcriptome (Table 1). The full-length cDNA sequence of these two contigs exhibited homology to NPF from *Nasonia vitripennis*. The precursor of the neuropeptide F-like transcript in *B*. *neritina* (BnNPFL) contains 116 amino acids, among which the first 25 amino acids are the predicted signal peptide. The mature BnNPY contains 35 amino acid residues (Fig 6, Table 2). Alignment of the mature BnNPFL with homologs from mollusks, insects and vertebrates revealed that the two tyrosine residues that are known to undergo sulfation are conserved in the putative BnNPFL peptide. However, the putative BnNPFL peptide was unique; the highly conserved C-terminal Arg^8^ residues within the RXRF motif were replaced with Pro^6^ (Fig 6). Neuropeptide Y/F or NPY-related peptides were collectively isolated from the central nervous system of both vertebrates and invertebrates [58–60]. The nervous system of *B*. *neritina* larvae is much simpler than that of vertebrates and insects. Although there is no well-defined CNS in the larvae, the neural plexus, which appears to be the only integration center for all sensory inputs, is generally considered to be the CNS equivalent [2, 5]. It will be interesting to assess whether BnNPY peptide expression is restricted to the neural plexus or occurs throughout the whole neural network.

#### Vasopressin-type peptide

**Fig 7:**
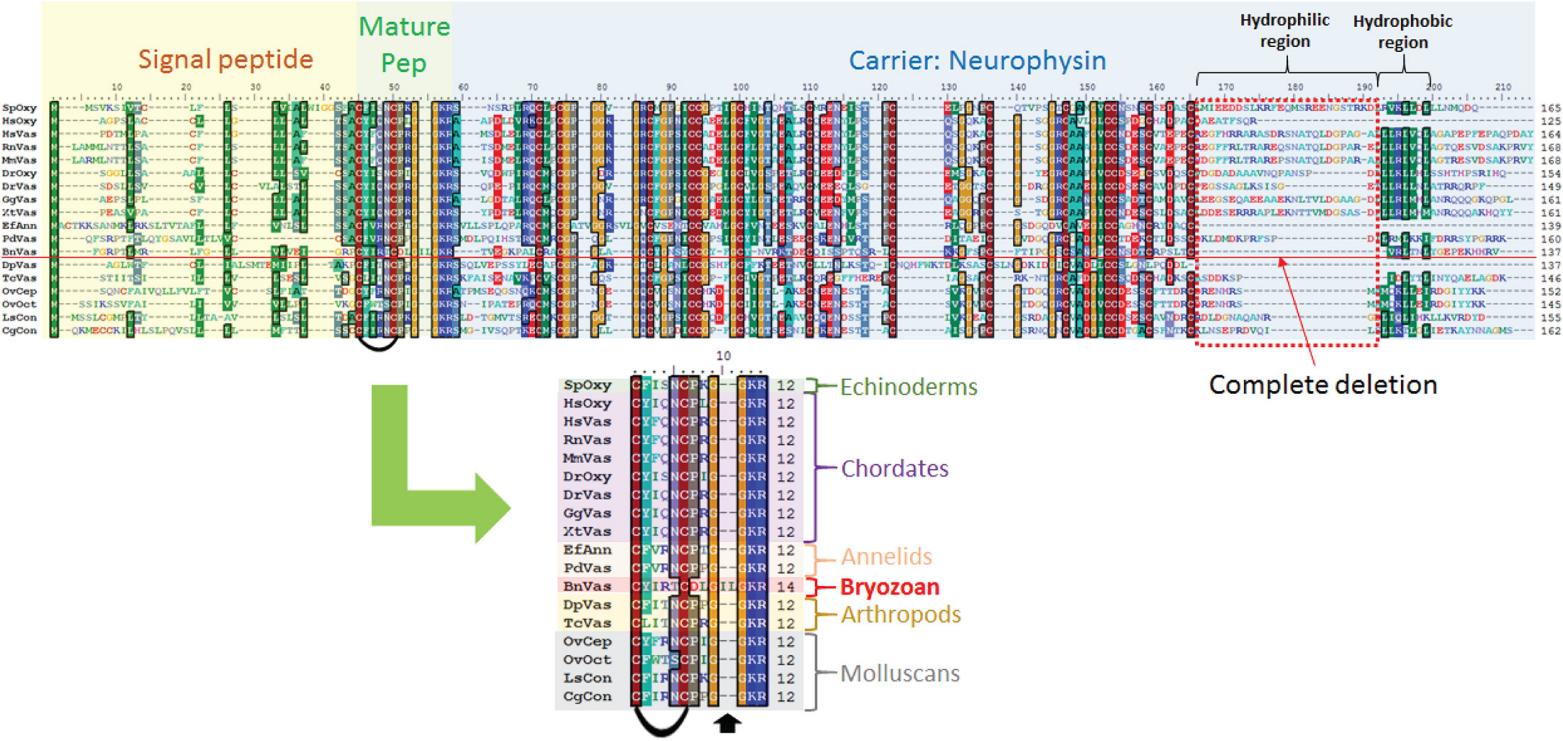
Multiple sequence alignment of BnVP-like with vasopressin and oxytoxin homologs, and VP-type peptide precursor from deuterostome and protostome taxa. SpOxy: oxytocin-neurophysin 1 [*Strongylocentrotus purpuratus*] (XP_003724490.1); HsOxy:) oxytocin-neurophysin 1 preproprotein [*Homo sapiens*] (NP_000906.1); HsVas: (P_000481.2) vasopressin-neurophysin 2-copeptin preproprotein [*Homo sapiens*]; RnVas: (CAA25795.2) vasopressin precursor [*Rattus norvegicus*]; MmVas: (AAC42027.1) neurophysin II/vasopressin [*Mus musculus*]; DrOxy: (NP_840076.1) oxytocin-neurophysin 1 precursor [*Danio rerio*]; DrVas: NP_840078.1| vasopressin-neurophysin 2-copeptin precursor [*Danio rerio*]; GgVas: (NP_990516.1) vasotocin-neurophysin VT precursor [*Gallus gallus*]; XtVaso: XP_002936404.1| PREDICTED: vasopressin-neurophysin 2-copeptin [*Xenopus (Silurana) tropicalis*]; EfAnn: (CAD20057.2) annetocin precursor [*Eisenia fetida*]; PdVas: (ABR68852.1) vasotocin-neurophysin [*Platynereis dumerilii*]; DpVas: (EFX71881.1) putative vasopressin-like neuropeptide preprohormone [*Daphnia pulex*]; TcVas: (EFA09262.1) arginine vasopressin-like peptide [*Tribolium castaneum*]; OvCep: (BAC84978.1) cephalotocin precursor [*Octopus vulgaris*]; OvOct: (BAC82435.1) octopressin [Octopus vulgaris]; (AAB35220.1) conopressin [*Lymnaea stagnalis*]; CgCono: (XP_011434624.1) PREDICTED: conopressin/neurophysin-like [*Crassostrea gigas*]. Disulfide bridges are indicated by a bolded curve. Two amino acids inserted in BnVP-like are indicated by the black arrow. The BnVP-like sequence is underlined in red. Note the hydrophobic region near the N-terminus of the putative carrier peptide neurophysin is completely absent in BnVP-like.

Vasopressin is one of the earliest characterized NPs. It was first discovered in the human pituitary gland [61]. Recent advancements in molecular biology have led to the discovery of vasopressin-type peptides in insects and crustaceans [62]. The vasopressin-oxytocin peptide family has a very ancient lineage and is structurally highly conserved from hydra to humans [63]. NPs within this family generally consist of 12 amino acids, with a disulfide bridge between the cysteine residues at positions 1 and 6 [61,64]. We recovered two transcripts that matched arginine-vasotocine from the *B*. *neritina* transcriptome database. Subsequently, we isolated the full-length transcript sequence of the precursor of this VP-type NP, which we labelled as BnVP-like. Multiple sequence alignment of the putative BnVP-like with oxytocin and vasopressin from both the protostome and deuterostome clades revealed a good correlation of cysteine motifs in both the carrier peptide neurophysin and the mature vasopressin-like peptide (Fig 7). The putative BnVP-like peptide is unique to other VP homologs in which the mature peptide consists of 14 rather than 12 amino acids, with two hydrophobic residues (Leu and Ile) in between the C-terminal Gly^4^ and Gly^3^. In addition, unlike vertebrate neurophysin homologs, the putative neurophysin peptide skipped the hydrophilic region between the first C-terminal cysteine and the conserved hydrophobic region (Fig 7). To our knowledge, this is the first report of a VP-type peptide in Bryozoa.

## Conclusion

We recovered the transcripts of 15 putative *B*. *neritina* NPs and peptide hormones by *in silico* transcriptome mining. Analysis of the mature structures and sequences of the predicted NPs provides the first description of bryozoan NPs. Most of the NPs identified in this work have obvious homologs in mollusks, insects and polychaetes. The spatial expression of three NP precursor transcripts (BnFDSIG-like, BnILP-B, BnGPB5) in *B*. *neritina* larvae was successfully characterized by *in situ* hybridization. Overall, the results of the present study extend our knowledge of peptidergic expression in bryozoans. Future comparative studies could focus on the shelled planktotrophic larvae that predated the non-feeding larvae in the evolution of gymnolaemate Bryozoa [65].

## Supporting Information

S1 Table RACE gene specific primers for candidate *B*. *neritina* NP precursor transcripts isolation. (DOCX)
