# Supplementary material for: In Silico Prediction of Neuropeptides/Peptide Hormone Transcripts in the Cheilostome Bryozoan Bugula neritina
Source: PLoS One. 2016 Aug 18;11(8):e0160271. doi: 10.1371/journal.pone.0160271 (PMC4990251; doi:10.1371/journal.pone.0160271)
Supplement: S1 Table — (DOCX) [file pone.0160271.s001.docx]

| Contig no. (From *B. neritina* transcriptome) | Precursor transcript name | GSP name | Nucleotide sequence (5'-3') |
| --- | --- | --- | --- |
| BN_contig_97286 | BnVP-like | AVS_3’RACE1 | TGGTGCTGGTGGAGATAGGA |
| BN_contig_87480 | BnVP-like | AVS_3’RACE2 | AGGACGTGTGATTTGGGCAT |
|  | BnVP-like | AVS_3’RACE3 | AACTAGACTAACTTCTGTTGC |
|  | BnVP-like | AVS_5'RACE1 | ACACCATCAATACAACTCTGCT |
|  | BnVP-like | AVS_5'RACE2 | GGGGGAGGAGATCTGACACT |
|  | BnVP-like | AVS_5'RACE3 | CTTCAGCTCACTCTACTGAACA |
| BN_contig_141741 | BnPEP-like | PEP_5’RACE1 | ACCCAAAAAGCGTTATCTGGAA |
| BN_contig_166562 | BnPEP-like | PEP_5’RACE2 | TGAGCAAATCGGCCATGGAA |
| BN_contig_98929 | BnPEP-like | PEP_5’RACE3 | ACACTACCCGATGAGTTGGC |
| BN_contig_220841 | BnFDSIG-like | LY_3’RACE1 | AAGCTGCACACTACTACGTTCA |
| BN_contig_55496 | BnFDSIG-like | LY_3’RACE2 | GCAATCCCCCTCATCCAGAG |
|  | BnFDSIG-like | LY_5’RACE1 | AAGGATCGTTTGAGTTCAATTTGT |
|  | BnFDSIG-like | LY_5’RACE2 | ACACTTTGCCAGCTCGGAAT |
| BN_contig_222301 | BnNPF-like | NPY_3’RACE1 | AGCTTTTTCGCCATAAATATCACA |
| BN_contig_184693 | BnNPF-like | NPY_3’RACE2 | TTGGCATACAACGACCCTGT |
|  | BnNPF-like | NPY_3’RACE3 | GGCTAACTTACTCAGACTATTTCTG |
|  | BnNPF-like | NPY_5’RACE1 | CCTACATTGAGAGACAATTCC |
|  | BnNPF-like | NPY_5’RACE2 | AGCCAATGGTACAAGAAGAATGA |
|  | BnNPF-like | NPY_5’RACE3 | TTACTCCACCCCTACCCCTC |
| BN_contig_4814 | BnGHB | GHB_3’RACE1 | AAGCTGCATACCTCAGAGCA |
|  | BnGHB | GHB_3’RACE2 | TGCTATGCGCAGTGGTTACT |
|  | BnGHB | GHB_3’RACE3 | AACGTCACTAACGGGGCAAA |
|  | BnGHB | GHB_5’RACE1 | TGGAGTGACTCATGGAGTGATTAT |
|  | BnGHB | GHB_5’RACE2 | CACTCTAGTCAGATGGGCCG |
|  | BnGHB | GHB_5’RACE3 | TTTGCGGCTTCGCTGACTAT |
| BN_contig_236733 | BnILP-D | MIRP3_3’RACE1 | GTACAACACAGTCACAGACAC |
|  | BnILP-D | MIRP3_3’RACE2 | CTGCACTGTGTACTTTCTGG |
|  | BnILP-D | MIRP3_3’RACE3 | CCAGCAGAGAAACAGCTAATGC |
|  | BnILP-D | MIRP3_5’RACE1 | AAGCTAGGCTATCAGCTGTAG |
|  | BnILP-D | MIRP3_5’RACE2 | GTGCTTGGATTCATCTGGAGA |
|  | BnILP-D | MIRP3_5’RACE3 | GGAGAGATTTGAGTTGAGCTTC |
| BN_contig_62648 | BnILP-A | INSA_3’RACE1 | CTTACATTCTGCTGGAATCAG |
| BN_contig_127297 | BnILP-A | INSA_3’RACE2 | AGCTTACAGCAGCTGTGGCTCT |
|  | BnILP-A | INSA_3’RACE3 | AGTACTGTGAGAGTAAGCGC |
|  | BnILP-A | INSA_5’RACE1 | GTCTAAAATGGGTTCACGCAGG |
|  | BnILP-A | INSA_5’RACE2 | GCATTCCTTATATCGTGGAAAG |
|  | BnILP-A | INSA_5’RACE3 | CATCTGCTTCATGGCGATGTTC |
| BN_contig_208700 | BnILP-E | ILP1_5’RACE1 | CCAAGGAAACTTTTCTCTCGTCA |
| BN_contig_236692 | BnILP-E | ILP1_5’RACE2 | TCAGCGCAAAGAAAGCCAAC |
| BN_contig_8730 | BnILP-F | MIRP_3’RACE1 | CCGTACATCAACAAGAGGGCA |
|  | BnILP-F | MIRP_3’RACE2 | TGCGCGAAGAAACGGAAAAG |
| BN_contig_203950 | BnILP-C | INSC_3’RACE1 | TCAAGATGGTTTCAGTGAGAATGC |
|  | BnILP-C | INSC_3’RACE2 | TATCACGGGCGAATGTCTCG |
|  | BnILP-C | INSC_5’RACE1 | CCTCTTGTAGAATCACTGAATCAGC |
|  | BnILP-C | INSC_5’RACE2 | TTGTGTGTGAATGCTGCGTG |
| Universial primer |  |  |  |
| oligodG(10)- adaptor primer | GGCCACGCGTCGACTAGTAC(g)10 | |  |
| oligodT(10)- adaptor primer | GGCCACGCGTCGACTAGTAC(T)17 | |  |
| T7 promoter-adaptor primer | TAATACGACTCACTATAGGGAGAGGCCACGCGTCGACTAGTAC | | |
| Adaptor primer | GGCCACGCGTCGACTAGTAC | |  |

Table S1. RACE gene specific primers for candidate *B. neritina* NP precursor transcripts isolation
